# Characterization of fluid occurrence states in shale reservoirs: centrifugal–nuclear magnetic resonance experimental analysis

**DOI:** 10.1098/rsos.250018

**Published:** 2025-08-06

**Authors:** Jin Pang, Tongtong Wu, Xinan Yu, Chunxi Zhou, Haotian Chen, Jiaao Gao

**Affiliations:** ^1^ Chongqing University of Science and Technology, Chongqing, People’s Republic of China

**Keywords:** shale reservoir, fluid occurrence state, centrifuge–nuclear magnetic resonance, gradient temperature drying method, methane adsorption experiment

## Abstract

The occurrence states of fluids in shale reservoirs directly influence the resource assessment of shale gas, reservoir permeability, selection of development technologies and economic benefits. Accurate analysis of fluid occurrence states is a key foundation for the efficient exploration and development of shale gas. To comprehensively elucidate the fluid distribution characteristics within shale pores, this study integrates centrifugation–nuclear magnetic resonance (NMR) experiments with stepwise thermal drying and methane adsorption analyses. By examining the NMR T₂ spectra of shale samples under varying centrifugal speeds, the distinction between movable and bound fluids is established, clarifying the influence of pore structure on fluid occurrence. Quantitative relationships between pore size and adsorbed/free gas are further investigated through methane adsorption experiments. Results demonstrate that centrifugation progressively removes water from macropores and microfractures, leaving residual water mainly confined to micropores. The stepwise thermal drying method efficiently differentiates movable water, capillary-bound water and clay-bound water. Integrating NMR analysis with methane adsorption reveals a significant impact of pore size on fluid occurrence: micropores predominantly store adsorbed gas, whereas macropores mainly contain free gas. These findings provide a theoretical basis for shale gas development and furnish essential data for optimizing exploration and production techniques.

## 1. Introduction

As a significant form of unconventional natural gas, shale gas plays a vital role in ensuring global energy security [1]. Shale reservoirs are characterized by low porosity and low permeability, with complex pore structures and diverse fluid occurrence states that directly influence gas accumulation and migration mechanisms [2]. A thorough understanding of the occurrence characteristics and distribution patterns of fluids in shale reservoirs, as well as their relationship to pore structure, forms the theoretical foundation for efficient shale gas development.

Accurate characterization of fluid occurrence states in shale reservoirs is key to evaluating gas content and production potential. Bustin *et al*. systematically categorized shale gas as existing in three primary states: free gas, adsorbed gas and dissolved gas [3]. Curtis found, via research on major North American shale gas basins, that adsorbed gas can account for 20–85% of the total gas content, with the proportion primarily controlled by organic matter abundance, maturity and pore structure [4]. Ross & Bustin further indicated that fluids within shales include not only gaseous phases but also bound and movable water, both of which significantly affect available storage space and gas mobility [5].

In recent years, nuclear magnetic resonance (NMR) technology has achieved significant advances in the characterization of fluid distributions within shales [6]. Chen *et al*. employed low-field NMR to investigate the spatial distribution of water in shale pores and established a quantitative relationship between the T₂ spectrum and pore size distribution [7]. Tinni *et al*. identified, through NMR experiments, that water in shales can be classified as clay-bound water, capillary-bound water and free water, with their relative abundance determined by mineral composition [8]. Cai *et al*. systematically analysed the occurrence of water in marine shale reservoirs and found that micropores (<2 nm) and mesopores (2–50 nm) are the main storage spaces for bound water, while macropores (>50 nm) predominantly contain movable water [9].

Accurately distinguishing fluid types in shale reservoirs is crucial for evaluating reservoir quality and informing development strategies. Loucks *et al*. classified shale pores as organic pores, inorganic pores and microfractures using scanning electron microscopy, with fluid occurrence characteristics differing significantly among pore types [10]. Clarkson *et al*. established a fluid classification system based on pore structure, highlighting that fluid behaviour in nanopores fundamentally contrasts with that in conventional reservoirs [11]. Studies on fluid mobility in shales have shown that the size of the pores critically determines the movability of contained fluids. Li *et al*., through centrifugation experiments, observed that pores larger than 100 nm facilitate greater fluid mobility [12]. Wang & Reed noted that movable fluids are mainly located in pores at the organic matter margins and within microfractures [13]. Yuan *et al*. discussed the impact of initial water saturation and water films on the spontaneous imbibition behaviour in tight reservoirs, demonstrating that water films can significantly reduce gas permeability [14]. Recent research indicates that the identification of fluid types must consider the integrated effects of various factors. Yang *et al*. reported that spontaneous imbibition in oil-saturated rocks is closely related to fluid type, wettability and pore structure, a finding that enhances our understanding of multiphase fluid interactions in shale reservoirs [15].

Accurate pore structure characterization provides a crucial basis for understanding fluid occurrence mechanisms [16]. Chalmers *et al*. proposed a multi-scale pore system characterization approach combining gas adsorption, mercury intrusion and NMR techniques [17]. Clarkson *et al*. introduced a fractal theory-based quantitative method for describing shale pore structure, revealing distinct fractal characteristics [18]. Regarding pore connectivity, Zheng developed a rapid evaluation method integrating spontaneous imbibition and NMR, enabling quantitative assessment of effective pore space and providing a new technical approach for evaluating the development potential of shale reservoirs [19]. NMR technology possesses unique advantages for characterizing pore structure. Washburn systematically reviewed NMR relaxation mechanisms and their applications in shale pore evaluation [20]. Bax & Lerner quantitatively analysed different fluids in shale pores using two-dimensional NMR measurements [21]. Zhang *et al*. established an NMR-based pore classification scheme, dividing pores into micropores (<10 nm), mesopores (10–100 nm) and macropores (>100 nm) [22].

The interactions between fluids and pore surfaces in shale reservoirs significantly influence fluid occurrence states. Javadpour first proposed the slip-flow mechanism of gases in nanopores, indicating that for nanometre-scale pores, the frequency of collisions between gas molecules and pore surfaces greatly exceeds intermolecular collisions, resulting in the breakdown of conventional Darcy flow [23]. Ambrose *et al*. found through molecular simulations that the thickness of gas adsorption layers in organic pores can reach several molecular layers, sharply reducing the effective flow space for gas [24]. Water–rock interactions are another vital factor impacting shale fluid occurrence. Dehghanpour *et al*. showed, via spontaneous imbibition experiments, that the hydration of clay minerals causes significant changes in pore structure [25]. Binazadeh *et al*. pointed out that water molecules form multilayer adsorption on clay surfaces, with thickness dependent on clay mineralogy and ionic strength [26]. Yang *et al*. found that temperature and pressure shifts alter the adsorption–desorption equilibrium of water, thereby affecting fluid occurrence states [27].

Despite recent advances [28,29], several key questions remain: (i) the distribution patterns of various fluid types within complex pore systems need further clarification, (ii) the quantitative relationship between fluid mobility and pore structure remains to be established, and (iii) the interaction mechanisms among coexisting fluids require additional research. Therefore, this study aims to investigate the occurrence characteristics of fluids in shale reservoirs using integrated centrifugation–NMR, stepwise thermal drying and methane adsorption experiments. Through quantitative analysis of fluid distribution in different pore types and construction of the relationships between pore structure and fluid states, this work seeks to provide new theoretical insights and technical support for efficient shale gas development. The innovations of this study are: (i) the establishment of a quantitative fluid classification and evaluation method based on centrifugation–NMR, (ii) revelation of the controlling mechanisms of pore structure on fluid mobility, and (iii) elucidation of the distribution patterns of adsorbed and free gas within different pore types. These findings offer significant theoretical implications and practical value for optimizing shale gas recovery and enhancing production efficiency.

The remainder of this article is structured as follows. Section 2 details the experimental materials and methods, including sample preparation, centrifugation–NMR procedures, stepwise thermal drying and methane adsorption experiments. Section 3 presents the results, such as NMR T2 spectra evolution under centrifugal forces and methane occurrence patterns. Section 4 discusses the mechanisms of fluid occurrence in shale reservoirs and the relationship between pore size and gas states. Finally, §5 summarizes the key conclusions and their implications for shale gas development.

## 2. Experimental material and methods

### 2.1. Sample preparation

In this study, four shale samples (designated R203-2, H202-2, Z201-1 and Z202-2) were collected from different depths of shale gas production wells. The basic petrophysical properties of these samples are as follows: sample R203-2 exhibits a saturated water porosity of 8.24% and is dominated by organic pores and fractures; sample H202-2 displays a saturated water porosity of 7.86% and presents a hybrid pore–fracture system of organic and inorganic origin; samples Z201-1 and Z202-2 both show a saturated water porosity of 3.96%, with inorganic pores and fractures as the primary pore system.

All shale samples were processed following standardized procedures. The original core was cut into cylindrical specimens (Ø25 ± 0.5 mm × 50 ± 1 mm) using a coring drill. To investigate the scale effect of samples on fluid migration characteristics, a portion of the cylindrical cores was mechanically crushed and sieved to obtain granular samples with particle sizes ranging from 20 to 40 mesh (0.425–0.850 mm). All specimens were oven-dried at 105°C for 24 h until constant weight was achieved, thereby removing any indigenous water. The dried samples were then subjected to vacuum saturation for 72 h using a 30 g l^−1^ KCl brine under vacuum conditions. The KCl solution effectively suppresses clay swelling and ensures the preservation of the original pore structure [30]. The vacuum saturation process guarantees that the pore spaces of the shale samples are fully saturated with brine, achieving the required initial water saturation for experiments.

### 2.2. Centrifugation–nuclear magnetic resonance experiments

NMR technology characterizes fluid distribution in porous media by monitoring the relaxation behaviour of hydrogen nuclei in a magnetic field. Once shale samples are placed in a homogeneous magnetic field, the hydrogen nuclei in the pore fluid are polarized. After the application of radiofrequency pulses, the nuclei return from the excited to the equilibrium state, and the transverse relaxation time (T₂) reflects the degree to which fluid molecular motion is restricted [31].

The relationship between T₂ relaxation time and pore size can be described as follows:

(2.1) $$\frac{1}{T_{2}}=\rho_{2}\frac{S}{V},$$

where *ρ*_₂_ is the surface relaxivity (μm ms^−1^) and *S*/*V* is the specific surface area of the pore space (μm⁻¹).

According to the range of T₂ values, the NMR T₂ spectrum of shale can be categorized into three characteristic intervals: a short relaxation region (T₂ < 10 ms) corresponding to bound fluids in micropores (<5 nm), an intermediate region (T₂ = 10–100 ms) corresponding to capillary-bound water in mesopores (5–100 nm) and a long relaxation region (T₂ > 100 ms) representing free fluids in macropores and microfractures (>100 nm) [32].

The NMR porosity (*ϕ*_NMR_) is derived from the NMR measurements and calculated using

(2.2) $$\phi_{NMR}=\frac{V_{fluid}}{V_{bulk}}\times 100\%=\frac{S_{total}\cdot K_{cal}}{V_{bulk}}\times 100\%,$$

where $\phi_{NMR}$ is the NMR-derived porosity (%), *V*_fluid_ is the volume of pore fluid, *V*_bulk_ is the bulk rock volume, *S*_total_ is the total T₂ spectral signal and *K*_cal_ is the instrument calibration coefficient. Unlike traditional helium porosity, NMR porosity only reflects the pore volume containing hydrogen-bearing fluids, excluding desiccated or non-hydrogen pores [33].

The variable-speed centrifugation method was used to simulate the stepwise expulsion of fluids from different pore types within shale. Five centrifugation speeds were set (4000, 6000, 8000, 10 000 and 12 000 r.p.m.), each run for 60 min. By measuring the change in sample mass and analysing the evolution of NMR T₂ spectra under different centrifugation conditions, the correspondence between pore type and fluid occurrence status was quantitatively assessed. NMR measurements employed the Carr–Purcell–Meiboom–Gill (CPMG) pulse sequence. Instrumental parameters were set as follows: resonance frequency, 2 MHz; echo interval (TE), 0.2 ms; number of echoes, 8000; number of scans, 64; waiting time (TW), 3 s; T₂ inversion range, 0.01–10000 ms (100 logarithmically spaced points). The original echo data were processed using a multi-exponential inversion algorithm to obtain the T₂ relaxation time distribution of the samples.

### 2.3. Temperature-gradient drying method

The stepwise thermal drying method exploits the differences in evaporation temperatures among various states of water, enabling the classification and characterization of pore water in shale through programmed heating and simultaneous NMR monitoring. The temperature ranges for removing different types of water are as follows: movable water (<80°C) primarily resides in macropores and microfractures; capillary-bound water (80–150°C) is held by capillary forces in mesopores and small pores; clay-bound water (150–200°C) exists as an interlayer or adsorbed water associated with clay minerals.

Saturated shale samples were placed in a precision temperature-controlled oven and subjected to staged drying following the temperature gradients: 80, 100, 120, 150, 180 and 200°C. Each temperature plateau was maintained for 4 h to ensure equilibrium in water evaporation. After each interval, samples were cooled to room temperature before immediate NMR T₂ spectrum analysis.

By comparing the evolution of T₂ spectra after drying at different temperatures, the relative content of each type of water was quantitatively assessed. The calculation is expressed as

(2.3) $$W_{i}=\frac{S_{T_{i-1}}-S_{T_{i}}}{S_{0}}\times 100\%,$$

where *W*_*i*_ is the percentage of water removed during the *i*th temperature interval, $S_{T_{i-1}}$ and $S_{T_{i}}$ represent the NMR signal intensity following drying at temperatures $T_{i-1}$ and *T_i_*, respectively, and S_0_ denotes the initial NMR signal under saturated conditions.

Based on the changes in peak positions and signal attenuation in T₂ spectra, together with mass loss data, the distribution characteristics of different water occurrence states can be reliably identified.

### 2.4. Methane adsorption experiment

Methane adsorption experiments were conducted using a combined NMR and gravimetric technique to construct methane adsorption isotherms [34]. The NMR signal intensity (*S*) is proportional to the number of hydrogen nuclei in the sample, and given that each CH₄ molecule contains four hydrogen atoms, the mass of adsorbed methane can be calculated as follows:

(2.4) $$m_{CH_{4}}=\frac{S\cdot M_{CH_{4}}}{4\cdot N_{A}\cdot K},$$

where *S* is the NMR signal intensity, *M*_CH4_ is the molar mass of methane (16.04 g mol^−1^), *N*_A_ is Avogadro’s number and *K* is a calibration constant determined by standardized samples of known methane mass [35].

The adsorption behaviour of methane was described using the modified Langmuir–Freundlich model:

(2.5) $$n_{ads}=\frac{n_{m}\cdot b\cdot P^{1/n}}{1+b\cdot P^{1/n}},$$

where *n*_ads_ is the amount adsorbed (mol g^−1^), *n*_m_ is the maximum adsorption capacity (mol g^−1^), *b* is the equilibrium constant (MPa⁻¹), *P* is the pressure and *n* is the heterogeneity parameter characterizing adsorption site energy distribution.

The content of free gas was calculated based on the real gas equation of state:

(2.6) $$n_{\text{free}}=\frac{PV}{ZRT},$$

where *V* is the pore volume (cm³ g^−1^), *Z* is the compressibility factor (calculated via the Peng–Robinson equation), *R* is the universal gas constant (8.314 J mol^−1^ K^−1^) and *T* is the experimental temperature (K).

Experimental procedures were as follows: samples were dried in a vacuum at 105°C for 24 h to constant mass. After loading into a high-pressure adsorption chamber, the chamber was evacuated to 10⁻³ Pa before introducing high-purity methane (99.999%) at specified pressure intervals (2.80, 4.86, 8.02, 10.76, 12.71, 14.62 MPa), with each pressure point allowed to equilibrate for 12 h at a controlled temperature of 30 ± 0.1°C. NMR T₂ spectra were recorded at each equilibrium pressure, and changes in the position and area of spectral peaks were used to distinguish between adsorbed and free methane.

The state of methane was determined by analysing the linearity of mass versus pressure relationships: a logarithmic relationship (*R*² > 0.95) indicates primarily adsorbed gas, while a linear relationship (*R*² > 0.98) is characteristic of free gas.

## 3. Results

### 3.1. Saturated water and NMR T_2_ spectra at different centrifugal speeds

The NMR T₂ spectrum quantitatively characterizes the fluid occurrence states in shale pores by analysing variations in peak positions and areas, with T₂ values correlating positively with pore size and peak area reflecting the fluid content in corresponding pores.

**Figure 1.**
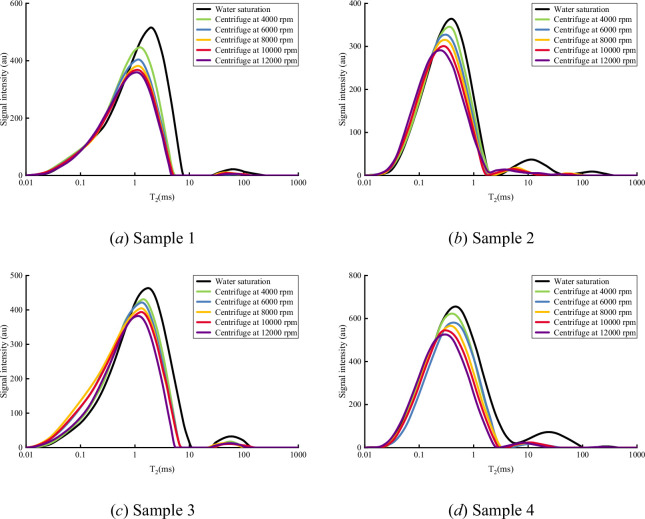
NMR T₂ spectra of saturated water and centrifugal experiments at different centrifugal speeds.

Figure 1 presents the evolution of T₂ spectra for four shale samples under water-saturated conditions and across a centrifugal speed gradient (4000–12 000 r.p.m.). In the saturated state, all samples exhibit a characteristic bimodal distribution:

The primary peak (T₂ < 10 ms), accounting for 65–75% of the total signal, corresponds to bound water in micropores (<5 nm).

The secondary peak (T₂ = 20–300 ms), comprising 25–35% of the signal, relates to movable water in macropores and microfractures (>100 nm).

As the centrifugal speed increases, the T₂ spectra display the following trends:

(1) *Peak shift*. The main peak shifts leftwards from 8.5 to 3.2 ms, indicating a reduction in average relaxation time corresponding to preferential expulsion of fluids from larger pores.
(2) *Peak evolution*. The intensity of the long relaxation peak (T₂ > 100 ms) decays exponentially. When the rotation speed reaches 8000 r.p.m., this peak signal drops to 15% of its original value; at 12 000 r.p.m., it is nearly extinguished (<2%), indicating that most movable water in macropores and microfractures has been expelled.
(3) *Retention of short-relaxation components*. The signal retention in the T₂ < 10 ms region remains high (72–85%), confirming that bound water in micropores is strongly constrained by capillary forces and cannot be readily removed through centrifugation.

The samples with different dominant pore types show clear differences in their response to centrifugation: organic pore–fracture systems (R203-2) exhibit the fastest long T₂ peak decay, indicating better pore connectivity, while inorganic pore–fracture systems (Z201-1 and Z202-2) show a higher proportion of short relaxation components, reflecting a pore system dominated by micropores.

### 3.2. Porosity changes and fluid distribution

**Figure 2.**
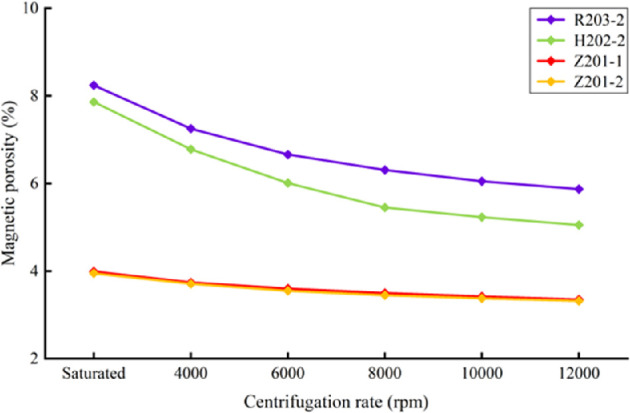
Changes in core sample NMR porosity at different centrifugal speeds and saturation states.

Figure 2 illustrates the evolution of NMR-derived porosity for the four shale samples subjected to various centrifugation speeds. The experimental results reveal a nonlinear decrease in NMR porosity with increasing centrifugation speed and highlight pronounced differences among samples with distinct pore types.

Under initial water-saturated conditions, the porosity values are as follows: organic pore–fracture type (R203-2), 8.24%; hybrid organic–inorganic pore–fracture type (H202-2), 7.86%; and inorganic pore–fracture type (Z201-1 and Z202-2), 3.96%. After centrifugation at 12 000 r.p.m., the porosities of the samples are reduced to 5.87% for R203-2 (28.8% decrease), 5.05% for H202-2 (35.8% decrease) and 3.33% for Z201-1 and Z202-2 (15.9% decrease). The magnitude of porosity reduction positively correlates with the initial porosity, indicating that samples with higher initial porosity possess more movable fluids.

The slope changes in the relationship between centrifugation speed and porosity allow the fluid expulsion process to be divided into three stages:

*Rapid expulsion stage (0–6000 r.p.m.).* The highest rate of porosity decrease is observed, mainly due to the expulsion of free water from macropores (>100 nm) and microfractures, accounting for approximately 65% of the total expelled fluid.

*Transition stage (6000–10 000 r.p.m.).* The rate of porosity reduction slows (0.18% per 1000 r.p.m.), corresponding to the expulsion of capillary-bound water from mesopores (10–100 nm), which constitutes about 25% of the total fluid loss.

*Plateau stage (>10 000 r.p.m.)*. The porosity stabilizes (decrease <0.05% per 1000 r.p.m.); strongly bound water in micropores (<10 nm) remains largely retained, with residual porosity comprising 64–84% of the initial value.

These results demonstrate that high-porosity samples harbour greater quantities of easily recoverable fluids, while low-porosity, micropore-dominated samples retain a larger proportion of bound fluid following high-speed centrifugation. The evolution of porosity and fluid expulsion profiles elucidates the intrinsic link between pore structure and fluid mobility, underscoring the critical role of pore architecture in shale gas recovery efficiency.

### 3.3. Effect of different pore types on fluid occurrence

To quantitatively assess the control of pore structure on fluid occurrence states, comparative experiments were conducted using both core plug samples (Ø25 mm × 50 mm) and crushed samples (20–40 mesh). This experimental design effectively elucidated several key mechanisms: (i) differences in fluid migration pathways—fluids in core plugs must traverse complex pore networks over longer distances to be expelled, whereas in crushed samples, the migration paths are significantly shortened, enabling evaluation of the impact of pore connectivity on fluid mobility, (ii) boundary effects—crushed samples possess greater specific surface areas, allowing fluids to escape more readily from grain boundaries, which can help identify fluids that are difficult to mobilize under original reservoir conditions but may be produced during field development, and (iii) simulation of different production scenarios—core plugs represent undisturbed reservoir conditions, while crushed samples mimic the behaviour of hydraulically fractured reservoirs and comparison between the two helps predict the enhancement of fluid recovery due to stimulation.

**Figure 3.**
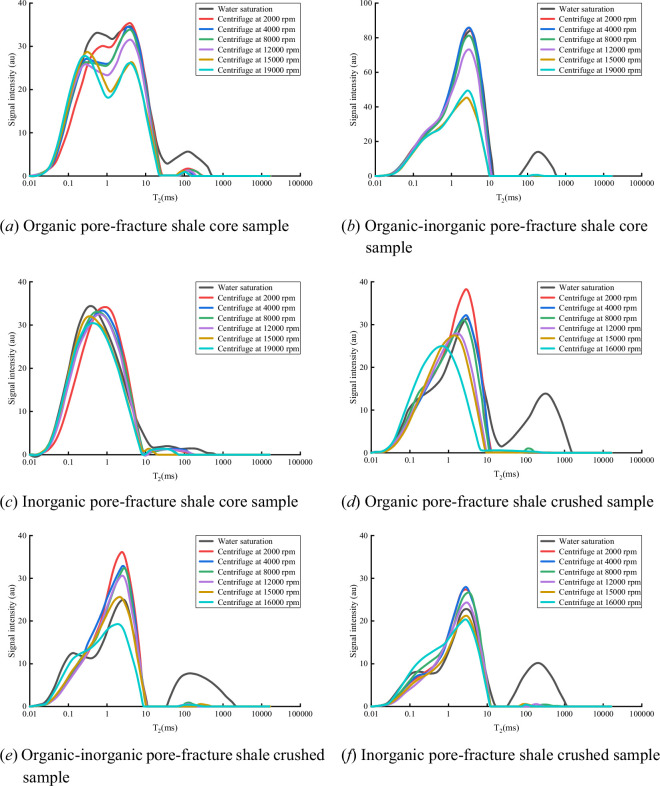
NMR T₂ spectra after centrifugal experiments for shale samples.

Figure 3 displays the evolution of NMR T₂ spectra for shales with different pore–fracture configurations under variable-speed centrifugation. The results show that the long relaxation peak (T₂ > 100 ms) diminishes markedly with increasing centrifugation speed, reflecting the preferential expulsion of movable fluids from macropores and microfractures. The long T₂ peak disappears completely in core plugs only at 15 000 r.p.m., whereas, in crushed samples, the same effect is achieved at merely 2000 r.p.m., indicating that shorter fluid migration pathways substantially reduce the driving force required for fluid expulsion.

Quantitative analysis based on NMR porosity further reveals significant differences in fluid mobility among different pore types:

*Organic pore–fracture type shale (R203-2)*. The porosity of the core plug decreases from 8.24 to 5.87% (a reduction of 28.76%) after centrifugation at 19 000 r.p.m.; for the crushed sample, the NMR signal intensity decreases from 1890 to 1192 AU (a reduction of 36.93%), with the proportion of movable fluid being 8.17% higher than that in the core plug.

*Hybrid organic–inorganic pore–fracture type shale (H202-2)*. The porosity of the core plug drops from 7.86 to 4.44% (a reduction of 35.86%); crushed samples show a decrease in signal intensity from 1423 to 904 AU (a reduction of 32.41%), with a 3.45% increase in movable fluid proportion.

*Inorganic pore–fracture type shale (Z201-1, Z202-2)*. The porosity of the core plug decreases from 3.96 to 3.33% (a reduction of 15.15%); for crushed samples, the signal intensity drops from 1257 to 985 AU (a reduction of 21.66%), corresponding to a 6.51% higher proportion of movable fluid.

These findings indicate that as particle size decreases, fluid migration pathways become shorter and the proportion of movable fluid during centrifugation increases. Therefore, by increasing centrifugal force or reducing particle size, even pore fluids that are initially difficult to mobilize can exhibit substantial flow capacity. In addition to highly mobile free water, significant volumes of capillary-bound water—previously considered immobile—may also be released and produced during reservoir development. These insights emphasize the fundamental influence of pore structure and connectivity on fluid occurrence and recoverability in shale reservoirs.

### 3.4. Methane adsorption and fluid occurrence states

**Figure 4.**
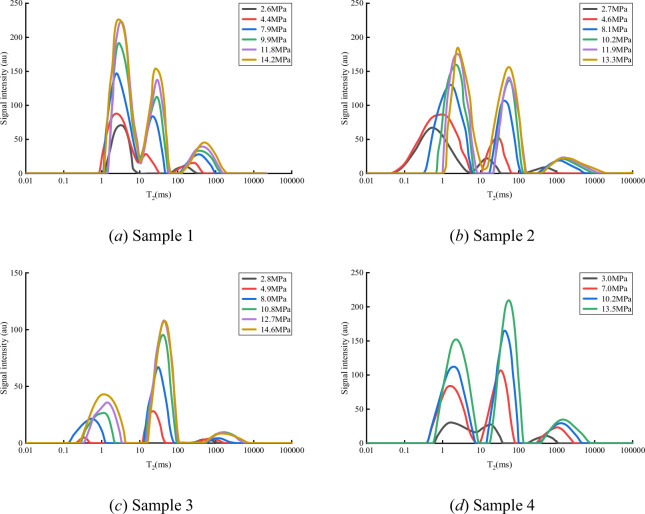
Methane adsorption NMR T₂ spectra under different pressure conditions for shale samples.

Figure 4 presents the NMR T₂ spectra for methane adsorption in the four shale samples under varying pressure conditions (2.80–14.62 MPa). All samples demonstrate a characteristic three-peak distribution, each with distinct physical significance:

*First peak (T₂ < 3 ms)*. Represents strong adsorbed methane, primarily residing in micropores (<5 nm), where methane molecules exhibit strong interactions with pore surfaces.

*Second peak (T₂ = 3–30 ms)*. Represents a combination of weakly adsorbed and free methane, mainly occurring in mesopores (5–100 nm).

*Third peak (T₂ > 30 ms)*. Corresponds to free methane, predominantly distributed in macropores and microfractures (>100 nm).

Analysis of peak areas shows that the first and second peaks account for 85–92% of the total signal, constituting the main storage space for shale gas. Although the third peak comprises only 8–15%, it plays a significant role in gas transport.

**Figure 5.**
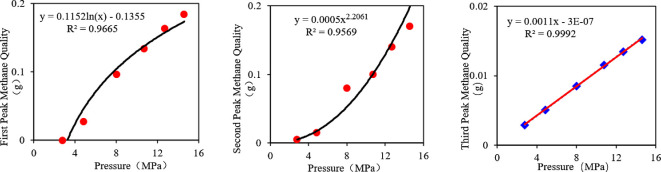
The relationship between the methane mass associated with each peak and pressure.

The relationship between NMR signal intensity and pressure (equation (2.4)), in conjunction with the equation of state, allows quantitative calculation of the methane mass associated with each T₂ spectrum peak. The calculation procedure is as follows:

*Calibration of signal intensity*. Establish a calibration curve of NMR signal intensity versus methane mass using standard samples with known methane content to determine the instrument calibration constant, *K*.
*Peak fitting*. Apply multi-Gaussian fitting to each T₂ spectrum at different pressures to recover the integrated area (signal intensity) for each peak.
*Quantitative calculation*. Convert the signal intensity of each peak to the corresponding methane mass based on the established calibration relationship.
*Adsorption state identification.* Examine the relationship between methane mass and pressure, as shown in figure 5.

The fitting results show:

*First peak.* Methane mass exhibits a logarithmic relationship with pressure (*R*^2^ = 0.9665)

(3.1) $$m_{1}=a_{1}\ln\left(P\right)+b_{1},\;R^{2}=0.9665.$$

*Second peak.* Methane mass shows a power-law relationship with pressure (*R*² = 0.9569)

(3.2) $$m_{2}=a_{2}\cdot P^{n2},\;R^{2}=0.9569.$$

*Third peak*. Methane mass and pressure maintain a linear relationship (*R*² = 0.9992)

(3.3) $$m_{3}=a_{3}P+b_{3},\;R^{2}=0.9992.$$

Here, *m* is the methane mass (mg g^−1^), *P* is the pressure (MPa) and *a* and *b* are fitting parameters.

Correlation analysis indicates that the logarithmic fit of the first peak (*R*² = 0.9665) is significantly higher than that of the second peak (*R*² = 0.9569), confirming that the first peak primarily corresponds to adsorbed methane. The strong linear correlation for the third peak (*R*² = 0.9992) demonstrates the typical characteristics of free methane. The power-law relationship observed for the second peak suggests a mixed adsorption-free state.

### 3.5. Fractal analysis of pore structure and gas content

By applying fractal theory in conjunction with NMR T₂ spectra, the pore system of shale was quantitatively divided into four categories [17]. According to the conversion between T₂ relaxation time and pore size (equation (2.1)), the threshold values for each pore type were determined as follows: micropores (T₂ < 3 ms, corresponding to pore sizes <5 nm), mesopores (T₂ = 3–30 ms, 5–100 nm), macropores (T₂ = 30–300 ms, 100–1000 nm) and microfractures (T₂ > 300 ms, >1000 nm).

**Figure 6.**
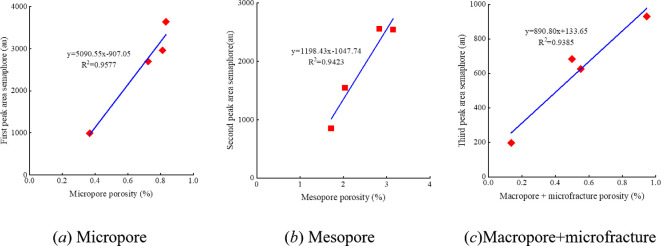
Relationship between pore volume and NMR signal intensity for each peak in the methane adsorption T₂ spectra after fractal analysis.

Peak integration of the T₂ spectra was used to calculate the volumetric fraction and corresponding porosity for each pore type. Figure 6 shows the correlation analysis between the porosity contributions of various pore types and the NMR signal intensities of each peak in methane adsorption T₂ spectra. It was found that the porosity of micropores exhibited a strong positive correlation with the area of the first peak in the methane adsorption T₂ spectra under dry conditions, with a correlation coefficient of 0.98. This indicates that the first NMR peak is mainly attributable to adsorbed methane in micropores. Similarly, the second peak is contributed by both inorganic and organic mesopores, while the third peak mainly reflects the influence of macropores and microfractures.

## 4. Discussion

### 4.1. Fluid occurrence mechanisms in shale reservoirs

This study reveals the intrinsic relationship between fluid occurrence states and pore structure in shale reservoirs, emphasizing that this interplay is a key factor influencing shale gas production efficiency. Shale reservoirs are characterized by highly complex pore networks comprising micropores, mesopores, macropores and microfractures, each exerting distinct control over fluid storage and mobility.

Results from the centrifugation experiments demonstrate that as the centrifugation speed increases, water present in larger pores and microfractures is progressively expelled, whereas fluids retained in micropores tend to be highly immobile and increasingly difficult to produce. Specifically, with higher centrifugal force, water in larger pores and microfractures is more readily removed, indicating that fluids in these domains chiefly exist in a mobile form and can be produced under relatively low driving forces. In contrast, fluids within micropores are subject to strong capillary forces and confinement effects due to their extremely small diameters, resulting in significant difficulty in liberation under standard conditions and the formation of capillary-bound water. This bound water fraction exerts a marked impact on shale gas deliverability. Yang *et al.* [36] employed molecular dynamics simulations to demonstrate that water exists in micropores (<5 nm) either in an adsorbed state or as capillary condensate, preferentially occupying high-energy adsorption sites. This results in a significant reduction in methane adsorption capacity with increasing water saturation.

The occurrence of fluids within micropores is closely associated with the formation of capillary-bound water. Because micropores typically have diameters less than 5 nm and possess high specific surface areas, water is likely to exist in an adsorbed or capillary-bound state. Capillary-bound water plays a vital role in shale gas reservoirs by not only restricting fluid mobility but also directly determining gas production potential. The persistence of capillary-bound water under normal conditions leads to the sequestration of a portion of the reservoir fluids, thereby decreasing shale gas recovery efficiency. Jiao *et al*. [37] employed molecular dynamics simulations and centrifuge–NMR experiments to reveal the layered distribution characteristics of water in nanopores (with adsorbed water density reaching 1.4877 g cm^−^³ and free water density at 0.9970 g cm^−^³), which aligns with the existence of water in micropores predominantly in adsorbed or capillary-bound states. The study further demonstrated that adsorbed water forms heterogeneous layers (approximately 0.63 nm in thickness) in pores smaller than 2 nm, leading to reduced gas diffusion capacity and confirming the blocking effect of capillary-bound water.

These insights are significant for understanding both the storage and migration behaviour of fluids in shale reservoirs. Particularly, strategies aimed at enhancing shale gas mobility and recoverability can benefit considerably from optimized methods for fluid expulsion. Thus, advancing the efficient release of bound water from micropores, mitigating capillary effects and improving fluid mobility within the finest pore spaces are critical challenges for enhancing shale gas production.

With technological advancements in shale reservoir stimulation, future research should focus on further reducing the adverse impacts of capillary-bound water through a combination of physicochemical methods (such as hydraulic fracturing and gas flooding) and novel fluid characterization techniques. Such efforts will inform the optimization of shale gas exploitation and support the sustainable development of the shale gas industry.

### 4.2. Relationship between pore size and adsorbed gas, free gas

Through fractal analysis of NMR T_2_ spectra, this study reveals that micropores primarily store adsorbed gas, while larger pores primarily store free gas. In shale reservoirs, micropores typically have pore sizes smaller than 5 nm and these small pores have a large specific surface area, enabling them to adsorb a large number of gas molecules. Adsorbed gas is primarily adsorbed on the surface of the pores in molecular form, influenced by strong capillary forces and intermolecular interactions. As a result, micropores are the main storage space for adsorbed gas in shale reservoirs. In these pores, the gas is usually in a stable adsorbed state and is difficult to release. It typically requires higher external energy input (such as increased pressure or temperature) to desorb and transform into free gas. The fractal dimension of the T₂ spectrum obtained after Liu’s centrifugation experiment (representing bound fluids) is higher than that in the saturated state, indicating a more complex micropore system (bound water/adsorbed gas regions). The fractal dimension of micropores shows a negative correlation with porosity and permeability, confirming that micropores dominate adsorbed-state storage (low permeability, high bound fluids). Furthermore, the concept of ‘macropores controlling storage capacity while micropores governing microscopic complexity’ is proposed in [38], which aligns with the aforementioned division of labour between adsorbed and free gases.

In contrast, larger pores (such as mesopores and macropores) primarily store free gas. The pore sizes of macropores are usually greater than 100 nm, providing sufficient space for gas to flow freely without being significantly influenced by capillary forces. Free gas molecules can exist independently in these pores and have higher mobility, allowing them to migrate and contribute to production under relatively low pressure gradients. Thus, larger pores play a key role in shale gas production, especially in the movement and extraction of shale gas, as they serve as the primary channels for gas transmission.

For the development of shale gas, understanding the relationship between pore structure and fluid occurrence is crucial. This relationship not only helps in accurately assessing the gas reservoir potential of shale reservoirs but also provides theoretical support for the optimization of development techniques. In particular, during the gas transmission and extraction processes in micropores and macropores, capillary effects and fluid dynamics play a key role. In micropores, the capillary effects result in stronger fluid retention, making it difficult to release the gas. In macropores, the gas has greater mobility and can migrate more easily through the pore network. Therefore, for reservoirs with different pore structures, development techniques must be tailored. For example, in reservoirs dominated by micropores, more refined hydraulic fracturing techniques may be needed to release bound gases, while in reservoirs dominated by larger pores, optimizing the gas transmission channels and enhancing gas mobility may be more important.

Overall, pore size has a profound impact on the storage and transmission characteristics of adsorbed gas and free gas. An in-depth study of the relationship between pore structure and gas occurrence will not only help us better understand the storage and migration processes of gas in shale reservoirs but also provide strong theoretical guidance for efficient shale gas development. In future studies, further integration of multi-scale characterization techniques for pore structure and gas flow models will help improve the accuracy and efficiency of shale gas extraction.

### 4.3. The innovation and limitations of the research

This study pioneers the integration of centrifugation–NMR coupled experiments, stepwise thermal desorption and methane adsorption analysis to achieve multi-scale quantitative characterization of shale fluid occurrence states: (i) by combining variable-speed centrifugation with NMR T₂ spectrum evolution, we established for the first time a quantitative correlation between pore types and fluid mobility; (ii) stepwise thermal desorption was employed to quantitatively distinguish mobile water, capillary-bound water and clay-bound water, clarifying their temperature response thresholds; and (iii) methane adsorption NMR spectra demonstrated the distribution patterns of adsorbed and free gas.

The experiments were conducted solely on shale samples from the Chongqing region and did not include typical reservoirs such as those with high clay mineral content or siliceous shale. The study focused on single-phase (water/methane) systems and did not account for interfacial effects under formation conditions when hydrocarbons and water coexist. Additionally, NMR T₂ spectra are unable to distinguish the adsorption layer thickness in pores smaller than 2 nm.

## 5. Conclusion

*Strong coupling between pore structure and gas occurrence state*. The pore size distribution in shale reservoirs directly governs the occurrence states of methane. Micropores serve primarily as storage sites for adsorbed gas, whereas larger pores predominantly accommodate free gas.

*Micropores as the dominant storage for adsorbed gas*. Due to their high specific surface area and pore size typically less than 5 nm, micropores adsorb a significant amount of methane. The gas in these pores exists mainly in the adsorbed state and is difficult to release under standard reservoir conditions.

*Large pores as reservoirs for free gas*. Mesopores and macropores, with pore sizes significantly greater than 5 nm, provide ample space for free gas storage. Methane molecules in these pores exhibit higher mobility and can be more easily produced during reservoir exploitation.

*Influence of capillary effects and kinetic energy on gas transport*. In micropores, pronounced capillary effects result in strong fluid confinement, making gas release challenging. Conversely, in macropores, the mobility of gas is greatly enhanced, facilitating efficient migration and production.

*Guidance for optimization of reservoir development strategies*. The variation in pore structure necessitates targeted development strategies for shale gas reservoirs. In formations dominated by micropores, advanced hydraulic fracturing may be required to enhance the release of adsorbed gas. For reservoirs with prevalent macropores, efforts should focus on optimizing transport pathways and improving gas mobility.

In summary, the occurrence state of methane in shale reservoirs is fundamentally controlled by the pore structure. A quantitative understanding of the interplay between pore architecture and fluid occurrence not only advances the scientific understanding of shale gas systems but also provides critical guidance for optimizing extraction technologies and improving recovery efficiency.

## Data Availability

All data included in this study are available upon request by contact with the corresponding author, or can be accessed online [39].
